# Preserved Renal Function in Kidney Transplantation over a Thrombosed Aortobifemoral Bypass Graft: The Role of Retrograde Flow and Early Thrombolysis

**DOI:** 10.1155/2016/6579591

**Published:** 2016-08-04

**Authors:** Saúl Pampa-Saico, Sara Jiménez-Alvaro, Fernando Caravaca-Fontán, Ana Fernández-Rodríguez, Maite Rivera-Gorrín, Juan Sánchez, Antonio Chinchilla, Roberto Marcén

**Affiliations:** ^1^Department of Nephrology, Hospital Universitario Ramón y Cajal, Carretera de Colmenar Viejo Km. 9.100, 28034 Madrid, Spain; ^2^Department of Medicine, Universidad de Alcalá, 28805 Madrid, Spain; ^3^Department of Radiology, Hospital Universitario Ramón y Cajal, 28034 Madrid, Spain; ^4^Department of Vascular Surgery, Hospital Universitario Ramón y Cajal, 28034 Madrid, Spain

## Abstract

Aortobifemoral bypass (ABFB) thrombosis is not uncommon, and when the artery of a renal graft is implanted on a bypass the risk of graft loss is high. We report the case of a 48-year-old woman with a previous history of ABFB under antiplatelet therapy and a kidney allograft implanted on the vascular prosthesis, who presented with acute limb ischemia and severe renal impairment. Imaging techniques revealed a complete thrombosis of the proximal left arm of the ABFB. However, a faint retrograde flow over the graft was observed thanks to the recanalization of distal left bypass by collateral native arteries. This unusual situation not previously reported in a kidney transplant setting, together with an early diagnosis, allowed graft survival until an early local thrombolysis resolved the problem. Two years later, renal function remains normal.

## 1. Introduction

Thrombosis of an aortobifemoral bypass (ABFB) graft is a common complication that may occur in about 10–15% of the cases within the first five years after surgery [1]. Moreover, when a renal allograft artery is implanted on a vascular prosthesis there is a higher risk of thrombosis and subsequent graft loss [2].

Here, we report the case of a patient presenting a complete occlusion of the arterial bypass in which renal graft perfusion was partially maintained by retrograde flow from collateral arteries in the distal end of the aortobifemoral bypass. Early diagnosis and preemptive treatment with local fibrinolysis allowed the resolution of thrombosis and complete recovery of kidney graft function.

## 2. Case Report

A 48-year-old woman with a previous history of chronic kidney disease secondary to type IV lupus nephritis (negative lupus anticoagulant and negative antiphospholipid antibodies) was diagnosed with Leriche syndrome in July 2012. Thus, a 14 mm × 7 mm expanded polytetrafluoroethylene (ePTFE) ABFB graft was successfully implanted and the patient was later discharged with antiplatelet therapy (acetylsalicylic acid 100 mg/day).

Four months later, the patient received a kidney transplant from a deceased donor. An end-to-side arterial anastomosis to the left branch of the ABFB was performed and an end-to-side venous anastomosis to the external iliac vein with no significant complications was performed. The patient was started on tacrolimus, mycophenolic acid, and steroids, reaching normal renal graft function over the next months (mean serum creatinine of 1 mg/dL).

Nine months later, the patient presented to the emergency room complaining of pain and coldness in the left lower limb and progressive decrease of diuresis in the last two days. Blood pressure was 185/105 mmHg, and physical examination revealed absence of popliteal and pedal pulses in the left lower limb and normal peripheral perfusion of the contralateral leg. Initial laboratory data revealed a serum creatinine of 4.1 mg/dL; lactate dehydrogenase 228 U/L (normal range: 140–240 U/L); creatine kinase 44 IU/L (normal range: 24–174 IU/L); hemoglobin 9.3 g/dL; leukocyte count 4.9 per 10^9^/L; serum tacrolimus concentration 4.6 ng/mL (reference range: 4–8 ng/mL). Urinalysis showed a sodium concentration of 55 mmol/L, with negative proteinuria and normal urinary sediment.

**Figure 1:**
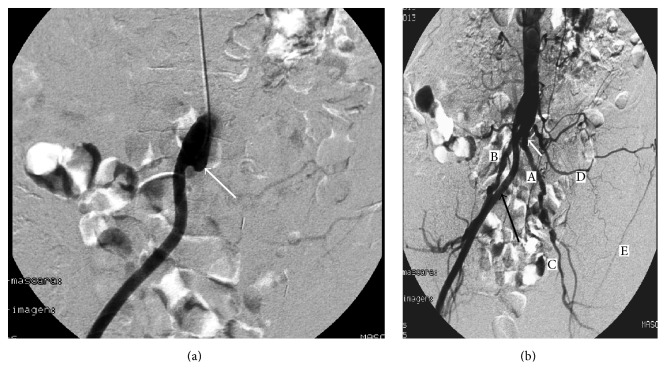
(a) Early image of abdominal angiogram showing a complete occlusion of the left arm of the ABFB (arrow). (b) The right arm of the ABFB is patent (black arrow); proximal button of the left ABFB (white arrow); primitive iliac arteries (A, B); collateral circulation: left hypogastric (C), left lumbar (D), and circumflex iliac (E) arteries.

**Figure 2:**
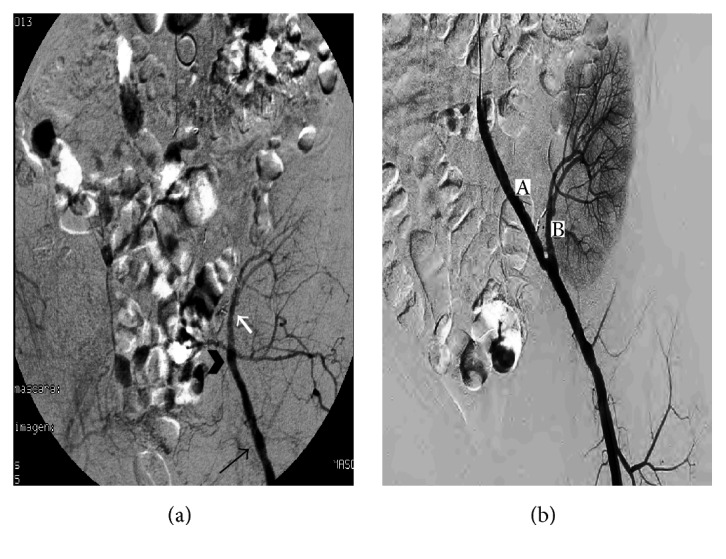
(a) Recanalization of both the distal branch of the left ABFB (head arrow) and renal artery of the graft (white arrow) and femoral common artery (black arrow). (b) Angiographic control showing both patency of the left arm of the ABFB (A) and the artery of kidney graft (B).

She was admitted to the hospital and an abdominal angiography performed through the right humeral artery revealed a complete thrombosis of the proximal left branch of the ABFB (Figure 1(a)), along with an important collateral circulation (Figure 1(b)). However, late-phase angiogram showed a retrograde filling of the distal prosthesis with only faint perfusion of the kidney (Figure 2(a)). Local fibrinolytic therapy with 10 mg of alteplase (recombinant tissue plasminogen activator-rtPA) was initiated, followed by systemic sodium heparin (250 IU/Kg) to achieve a target activated thromboplastin time between 1.5-fold and 2.0-fold over the normal range. A control angiogram 8 hours after that showed a successful restoration of patency of the bypass, together with both a normal nephrogram and arterial vascular tree of the kidney (Figure 2(b)).

The patient was discharged 14 days after admission under treatment with oral anticoagulation (acenocoumarol), with a complete resolution of ABFB thrombosis and recovery of kidney graft function (serum creatinine concentration of 1.2 mg/dL).

## 3. Discussion

This case report highlights the risk of development of acute renal allograft failure in kidneys transplanted over vascular grafts. A myriad of case reports documenting the recovery of renal function after thrombolysis have been published; however, to the best of our knowledge, this is the first case of a kidney allograft implanted on a vascular bypass graft.

Kidney transplantation on prosthetic vascular grafts was described first by Sterioff in 1974 [1], although it is not a frequent procedure nowadays according to case series (0.2–1.7% of renal transplants) [2, 3].

The etiology of vascular graft thrombosis may be related to progressive fibrosis and development of stenosis or aneurysms at the site of implantation which could eventually lead to abnormal vascularization [2–4].

Clinically, the presence of coldness and/or lower limb pain with no peripheral pulses in patients with ABFB suggests a thrombosis of the bypass graft. In our patient, the coexistence of reduced urine output pointed out that the perfusion of the kidney graft could also be compromised.

Peripheral arterial occlusions are usually associated with increased leukocyte count and increased serum levels of lactate dehydrogenase [5], although our patient had normal values. It has been reported that, in the setting of renal artery thrombosis, there may be a great variability in laboratory values depending on the number and size of segmental arteries affected, the intensity of ischemic injury, the time of development, and the presence of collateral circulation. As expected, thrombosis of the main renal artery in patients with only one functioning kidney is associated with impaired renal function. However, urinary parameters may vary depending on the stage of arterial occlusion [5, 6].

In our case, the patient had severe renal impairment and oliguria at presentation, but retrograde filling of the distal vascular graft permitted a partial perfusion of the transplanted kidney. This was probably the result of a high blood pressure in collateral arteries and relatively low intra-arterial resistance in renal graft with respect to the limb, and we believe that this phenomenon played a pivotal role in the allograft viability.

Finally, early recognition and initiation of thrombolytic therapy restore the circulation. We initiated therapy with rtPA due to the low side-effect profile and ease of use. Moreover, it is less aggressive than surgical thrombectomy, and it is associated with a lower average length of stay in hospital [7–9].

In conclusion, thrombosis of an ABFB with an implanted kidney allograft may not result in graft loss if early diagnosed and treated. The presence of collateral circulation together with retrograde filling of the vascular prosthesis played an important role in maintaining the viability of the organ.
